# Anthroengineering: an independent interdisciplinary field

**DOI:** 10.1098/rsfs.2020.0056

**Published:** 2021-08-13

**Authors:** Michael A. Berthaume, Patricia Ann Kramer

**Affiliations:** ^1^ Division of Mechanical Engineering and Design, London South Bank University, London SE1 0AA, UK; ^2^ Department of Anthropology, University of Washington, Seattle, WA 98195-3100, USA; ^3^ Department of Orthopaedics and Sports Medicine, University of Washington, Seattle, WA 98195-3100, USA

**Keywords:** anthroengineering, transdisciplinary, anthropology, engineering, biomechanics, biological anthropology

## Abstract

In recent decades, funding agencies, institutes and professional bodies have recognized the profound benefits of transdisciplinarity in tackling targeted research questions. However, once questions are answered, the previously abundant support often dissolves. As such, the long-term benefits of these transdisciplinary approaches are never fully achieved. Over the last several decades, the integration of anthropology and engineering through inter- and multidisciplinary work has led to advances in fields such as design, human evolution and medical technologies. The lack of formal recognition, however, of this transdisciplinary approach as a unique entity rather than a useful tool or a subfield makes it difficult for researchers to establish laboratories, secure permanent jobs, fund long-term research programmes and train students in this approach. To facilitate the growth and development and witness the long-term benefits of this approach, we propose the integration of anthropology and engineering be recognized as a new, independent field known as *anthroengineering*. We present a working definition for anthroengineering and examples of how anthroengineering has been used. We discuss the necessity of recognizing anthroengineering as a unique field and explore potential novel applications. Finally, we discuss the future of anthroengineering, highlighting avenues for moving the field forward.

## 1. Introduction

**Figure 1.**
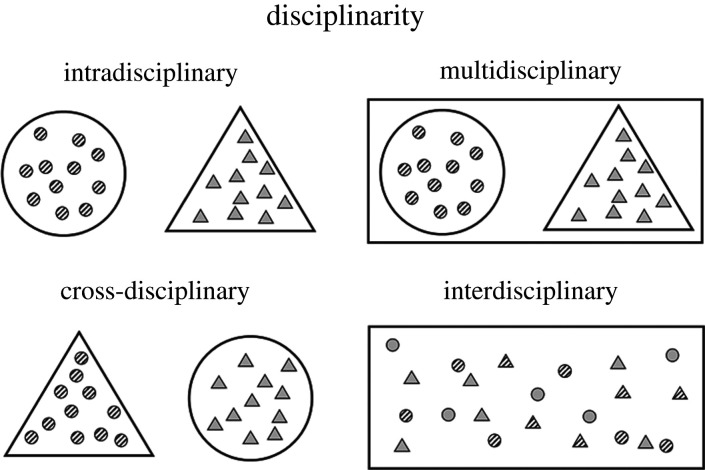
Types of disciplinarity that combine anthropology (circles) and engineering (triangles). Intradisciplinary: anthropologists (striped circles) and engineers (grey triangles) work within their respective fields (large circle and triangle). Multidisciplinary: anthropologists and engineers work within their respective fields to address a larger issue (rectangle). Cross-disciplinary: anthropologists investigate issues within engineering, and engineers investigate issues within anthropology. Interdisciplinary: anthropologists, engineers, anthropologists turned engineers (striped triangles) and engineers turned anthropologists (grey circles) seamlessly use both disciplines, simultaneously, to address larger issues.

Transdisciplinarity forms a common axiom that transcends the disciplines, creating an overarching synthesis [1] (figure 1). As these syntheses combine previously isolated thoughts and ideas, the knowledge created by their integration is greater than anything that can be created by a single discipline on its own. Simply put, the whole is greater than the sum of its parts (Aristotle). Here we propose a new field that transcends existing disciplines: anthroengineering.

**Figure 2.**
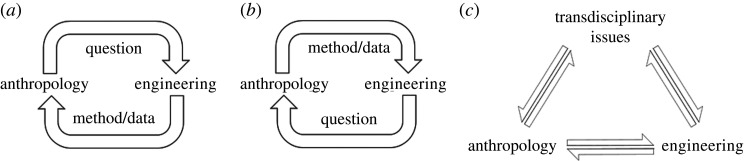
Transdisciplinary approaches to anthroengineering. (*a*) Engineering method(s)/data being leveraged to address anthropological question(s). Through an iterative process, question(s)/method(s) are refined and a synthesis is reached (e.g. the application of FE modelling to human evolution [10–13]). (*b*) Anthropological method(s)/data being leveraged to address engineering question(s). Through an iterative process, question(s)/method(s) are refined and a synthesis is reached (e.g. the application of ethnography to engineering design [14]). (*c*) Engineering and anthropological questions, methods and data are used to address transdisciplinary issues (e.g. design and/or manufacture of culturally relevant, sustainable medical devices for low- and middle-income countries).

A recent transdisciplinary trend combining anthropology and engineering—anthroengineering—has become increasingly popular over the last few decades. It has played a crucial role in the development of fields such as biomechanics [2,3], ergonomics [4,5] and functional morphology [6–9]. Anthropology—the science and study of human and societal culture, language and biology—and engineering—the application of science to create machines and implement technologies and tangible solutions to societal problems—are unique and distinct disciplines that infrequently share curricular overlap. When the transdisciplinary approach has been applied to anthropology and engineering, it has often leveraged methods or data from one discipline to address a question from the other (figure 2). This focus on specific problem-solving rather than a united theoretical foundation limits the impact of any innovations created by the collaboration. Thus, the power of the transdisciplinary approach is not fully realized. By leveraging both disciplines to address issues that transcend each discipline (i.e. transdisciplinary issues), syntheses can be created that are of interest not only to members of both disciplines, but also to individuals outside of either.

The uniqueness and distinctiveness of the two disciplines means that, if a Venn diagram were to be drawn, little overlap would be apparent. Thus, it is difficult for researchers to identify issues that simultaneously leverage both disciplines. Yet, such issues exist, and many of them are crucial for the success of people and planet. Examples of such issues include the United Nations (UN) 17 Sustainable Development Goals (SDGs). These goals set forth a blueprint for how to achieve a more sustainable future for all by addressing problems ranging from poor health to inequality, environmental degradation, and peace and justice [15–17]. Because anthropologists and engineers are trained to approach these problems in discipline-unique ways, their perspectives will be distinct along a multitude of axes, and the fusion of the two disciplines will be difficult. But, ultimately, the insights gained will lead to solutions that neither discipline could achieve independently.

Despite the presence of significant overlapping issues and great benefits that could be achieved by leveraging both anthropology and engineering to address these issues, this transdisciplinary approach is rare, because no generalized framework that incorporates anthropology and engineering currently exists. Instead, frameworks are constructed for targeted projects which are often abandoned when the project is completed. Establishment of these frameworks requires an extraordinary amount of effort, and their specificity and frequent abandonment prevents them from being used for novel applications. A generalized framework is needed.

Such a framework would require, among other attributes, a common language where anthropologists and engineers can communicate effectively. It would require acknowledgement, respect and integration of expertise to develop new syntheses and a new cohort of students who are trained to think as both anthropologists and engineers simultaneously. But before a framework can be developed, this transdisciplinary approach requires a name. Without a name, the approach remains unknown, ill-defined and abstract. But with a name, this approach has identity and carries with it symbolism beyond its meaning. We suggest, therefore, that the transdisciplinary approach, combining both anthropology and engineering, be recognized as its own, independent field called *anthroengineering*.

## 2. What is anthroengineering

Anthroengineering is an approach that uses theories, methods and/or data from both anthropology and engineering to address questions within and beyond both disciplines. The result is the development of new knowledge, which can take a multitude of forms (e.g. data, technologies, viewpoints, axioms, syntheses). While the true power of such an approach would lie in leveraging it to address transdisciplinary issues, anthroengineering can also be used to address questions within anthropology and engineering and to advance each field individually (figure 2).

Providing anthroengineering with a name, describing it and recognizing it as distinct entity allows for researchers to succinctly define their work and, more importantly, provides them with identity as anthroengineers. It also acts to provide a common thread and search term that can tie together all future work that uses a transdisciplinary approach to combine both anthropology and engineering. Doing so will provide those interested in anthroengineering with a direct way to learn about it and what frameworks, data and methods exist to leverage anthroengineering effectively in their own work.

## 3. Examples of anthroengineering

As previously discussed, examples of anthroengineering already exist, and some have existed for decades. Given our expertise, we discuss some examples largely through the lens of biological anthropology and engineering mechanics.

### 3.1. Classic anthropology meets classic engineering

Anthropologists have studied dental wear patterns on the micro-, meso- and macro-levels for over a century [18] to address a myriad of questions in such topics as taxonomy [19], palaeoecology [20], environmental reconstruction [21] and behaviour [22,23]. Similarly, mechanical failure analyses—and, in this situation, tribology and fracture mechanics—have been a major focus of engineering since the birth of the field as all machines experience wear [24–26]. It is, therefore, unsurprising that anthropologists and engineers have teamed up to understand better how teeth wear and fracture.

Using techniques such as nano-indentation, researchers have been able to investigate the role that microscopic particles (e.g. phytoliths, grit) play in the wear of dental enamel [27,28]. Additionally, through physical experimentation, modelling and comparative anatomy [29–32], researchers have been able to investigate the role of enamel thickness and schmelzmusters (enamel microstructure) in force and energy in failure resistance. Although researchers may not always agree on interpretations of experimental results [33–35], this research has led to advances in understanding dental wear and the factors that influence it [36], understanding functional adaptations of teeth [37,38] and the creation of bioinspired materials [39].

Similarly, principles from material science and solid mechanics (engineering) and musculoskeletal biology (anthropology) have been used to understand how skeletal form (shape + size) and skeletal and ecological mechanical properties affect the way loads are transferred to the skeleton and how the skeleton responds to internal and external loads. Bone (re)models in response to mechanical strain [40–43]: this in turn affects bone's mechanical properties (remodelling) and form (modelling) (e.g. [44,45]). Bone strains have been measured experimentally using *in vivo* [46,47] and *in vitro* [48,49] techniques using strain gauges and digital image/volume correlation (DIC, DVC). However, this only delivers information on bone strain at a limited number of sites. By constructing finite-element (FE) models and validating them using experimental strains [50,51], we can obtain three-dimensional strain maps across the entire structure.

FE models require several inputs, including geometry, constraints and mechanical properties [8,52–54]. Advances in three-dimensional scanning techniques, computer science and statistical shape modelling (e.g. geometric morphometrics [55], dental topography [56]) have made it possible to not only (re)construct three-dimensional digital representations of such models [9,57–61], but also quantify complex shapes for statistical analyses [55,62–64]. Constraints come from muscles, joints and/or the external environment. Muscle force can be estimated by multiplying maximum force generation—originally estimated using physiological cross-sectional area [65–67] but now relying on muscle activation/strength [68] and often validated using electromyography [69–71]. Joint constraints are estimated using anatomical knowledge and skeletal collections. Although constraints from the external environment are often modelled as reaction forces, the mechanical properties of the environment (e.g. ground substrate composition during locomotion [72] or dietary mechanical properties during mastication [73,74]) affect the rate and manner in which the load is transmitted. Finally, mechanical properties are difficult to obtain, as bone is a hierarchical, composite structure, but techniques such as tension/compression tests, bending, indentation and ultrasound are used to estimate static and dynamic (bulk) properties [75–82]. Sensitivity studies are useful in understanding how parameter estimates affect the results, but not in validating the model [49,83–86], which requires data from empirical studies (e.g. [53,83]).

Using an extensive array of theories and methods from anthropology and engineering, we have learned more about musculoskeletal biomechanics than can be listed here. Some major findings include:

(1) Over a lifetime, an individual will engage in actions that will load their skeleton. In turn, their bones will generate a set of mechanical properties and forms to properly resist the *in vivo* strains brought on by those loads [42,87–89]. But it can be difficult or impossible to determine what actions occurred in the lifetime of an individual given only a set of bone mechanical properties and forms, as multiple behaviours can yield similar loading regimes. This is further complicated with inter-populational or among-species comparisons, as genetics and neutral selection play a significant role in bone form [90].
(2) Skeletal morphologies particular to specific hominin species have focused attention on the relationships among form, function and behaviour [91]. For instance, the lower limb and pelvic morphology of *Australopithecus afarensis* (e.g. [92]), *Australopithecus sediba* (e.g. [93]) and *Homo neanderthalensis* (e.g. [94]) has led to long-term debates regarding their forms of terrestrial locomotion. Geometric morphometrics and other traditional statistical analyses have led to important insights (e.g. [95]), although they quantify skeletal form and not biomechanical function, and many questions remain. Inverse dynamic simulation of walking in extinct hominins offers the opportunity to expand our understanding of this critical behaviour (e.g. [92,96]), but the integration of musculoskeletal models offers the best opportunity for future insights [68].
(3) Masticatory loads cause mechanical strains in the skull, which significantly affect its mechanical properties and form [44,45,97]. However, the debate about the relationship between feeding mechanics and diet has led to major questions: is it possible, over an individual's lifetime, to develop a skull that is over- or under- designed for the masticatory loads it experiences [98,99]? Does a skull's ability to resist masticatory loads dictate or limit an animal's or species’ diet? Does natural selection select for skull form based on its ability to resist masticatory loads [10–13,100–102]?
(4) Primate tooth shape is undoubtedly correlated with diet [56,103], likely because teeth have evolved to break down foods consumed more efficiently [56,104,105]. However, the interactions between multicusped teeth and food items are so complex that we lack an efficient model for describing these relationships and, thereby, predicting food item breakdown from tooth shape [64,106,107].

Although it may seem that these lines of research have created more questions than answers, the independent syntheses of anthropology and engineering have led to important insights not only for the fields of anthropology and bioengineering but also anatomy, evolution, medicine and dentistry, to name a few. Further, the crucial questions generated would not exist if not for this transdisciplinary anthroengineering approach, and researchers would be ignorant of their ignorance.

### 3.2. Addressing intradisciplinary questions

Anthroengineering has also contributed in addressing more targeted questions within the disciplines of anthropology and engineering. Owing to decades of research in relatively independent fields, anthropology can provide insights into the Universe that engineering does not have, and vice versa.

Because anthropology is a discipline dominated by questions, while engineering a discipline that focuses on methods and applications, it is easy to see how the tools of engineering can be used to address anthropological questions. For example, using methods initially developed in engineering, virtual anthropology [108] has made it possible to quantitatively reconstruct palaeoarchaeological material and statistically quantify the accuracy of these reconstructions [59–61,109,110]. Two important examples of this are the reconstruction of the skull of *Ardipithecus ramidus*, which provided crucial, previously missing information about the evolution of hominin social structure, bipedalism and brain structure in hominin evolution during the Pliocene [110]. Additionally, the reconstruction of the mandible of *Homo habilis* not only showed a decoupling of brain and tooth size, but also allowed the development of a hypothesis regarding a much earlier origin of the genus *Homo* [109]. While that paper was under review, a new fossil (the Ledi-Geraru mandible) was discovered, confirming the authors' hypothesis [111].

Two additional common engineering methods—FE analysis and tension/compression tests—have been used extensively in palaeoanthropology to quantify the biomechanical performance of hard skeletal tissues and address questions concerning the evolution of primate diets [12,46,58,112–114]. The ability to print three-dimensional fossils further allows for the mechanical testing of previously inaccessible material [115–117]. These *in silico* and *in vitro* models and experiments carry with them several assumptions about the loading conditions and mechanical properties of the structure being analysed but provide valuable information about the biomechanical limits of the structure.

Given the plethora of methods in engineering, it may be more difficult to see how anthropology can benefit engineering. Nonetheless, engineering focuses on the application of science to solve problems for people, and anthropology is uniquely situated to provide the context to those problems. For instance, anthropology has improved engineering through the incorporation of anthropological methods. For example, the incorporation of ethnography into design to form the fields of design/techno-anthropology [14] and conferences like EPIC (Ethnographic Praxis in Industry Conference; www.epicpeople.org). End-user design focuses on the user's needs when designing products. By using anthropological techniques like ethnography, engineers can gather information about the wants and needs of the user that is inaccessible through focus groups developed from marketing perspectives. A classic example is in the design of the MP3 player, which was meant as an affordable alternative to the iPod to be used in the gym. Focus groups thought they wanted a device with many options and, therefore, many buttons. The product was designed, sent to market and failed. It was only by teaming up with ethnographers that designers and engineers realized that people's hands got sweaty in gyms and that gyms were social places. Ultimately, people actually wanted devices with fewer buttons and a quick on/off switch—they just did not realize it when they were in focus groups because the questions were not asked in the proper framework [118].

Anthropologists bring with them techniques that can be used to design for the future [119] and understand the consequences of technological advances. Engineers make design decisions to help today, but rarely think about the long-term effects on societies and communities in the future: this is because many work for companies which are on a deadline and, once one project is complete, they move on to the next. A classic example of the desire to solve the problem at hand without considering the potential longer-term societal consequences has been documented [120]. Engineers working through an international development organization created a solution to a chronic water shortage by developing a 140 km gravity-driven water pipeline that supplied water taps in local settings. Before the project, local women had carried water from natural sources, at times a journey of 3 h. The water distribution system worked well, but two unintended consequences occurred: the decrease in energy expenditure due to no longer needing to carry water increased the women's fertility and, because nutritional resources remained the same, increased child malnutrition [120]. These consequences are predictable through the lens of human reproductive ecology, a key body of knowledge in biological anthropology.

Anthropologists are trained to investigate long-term societal and community trends and are in a much better position not only to understand but also to address these problems. By working together, anthropologists and engineers who are interested in finding more socially connected solutions can do more to address crippling human problems. An example of how this can work came forth at the ‘Why the World Needs Anthropologists: Powering the Planet’ conference at Durham University, UK, in 2018. The conference focused on the problems facing energy (e.g. production, dissemination, storage) and explored how energy professionals and anthropologists can work together to create energy innovations that change the world for the better (https://www.dur.ac.uk/dei/events/?eventno=34503). In many cases, applied anthropology, which focuses on the external application of anthropology to current problems, could be used to extend and/or enhance the solutions to the problems engineers are regularly faced with.

Finally, although biomimicry is a field in itself, its application often falls short of its potential. Engineers who use biomimicry often look at the biological system in isolation and with overly simplified biological theories (e.g. assuming natural selection has caused a structure to be optimal for its function, without considering the evolutionary history of that element). Biological solutions typically must solve several simultaneous problems and have evolved within a set of allometric, phylogenetic and ontogenetic constraints [121]—a core understanding in biological anthropology—and the adaptationist programme frequently employed by engineers has been rejected by biologists for decades [122,123]. Because of this, biomimetic engineering falls short of its goals.

Anthropologists are trained to consider biological context that could lead to more effective biomimicry solutions using primates and human biological systems (e.g. the hierarchical structure of bone [124]). Take, for example, the design of the human foot, a complex structure that can be rigid in some circumstances and compliant in others. The evolutionary history of the foot is complex and filled with gaps [125], but we know it has evolved to interact with various substrates [72]. When wearing a shoe, the substrate interacting with the foot is no longer the ground, but the shoe itself [126], but shoe design does not often take foot–substrate interactions into account. Many shoe designs lead to running biomechanics that the human skeleton has not evolved to handle (e.g. high-impact forces during heel striking [127]). Similar issues can be seen in prosthetic foot design, where the impact of foot stiffness on gait biomechanics is well documented for advanced prosthetic feet (e.g. [128]). But in situations with fewer opportunities for the use of advanced medical devices, ‘one size fits all’ becomes ‘one stiffness fits all’ and the negative consequences of such choices are not appreciated. Further, even advanced medical interventions select a specified, unchanging stiffness for the prosthetic foot, when the natural foot has an adaptive, continuously changing stiffness, dependent on substrate and loading. Using anthroengineering and biomimicry approaches, answers to questions like ‘How can we use what we know about variation in Primates to make engineered products better?’ are achievable.

## 4. Why recognize a formal field of anthroengineering?

If anthroengineering projects already exist, why is it necessary to provide the word ‘anthroengineering’ to describe them all? It is not as if the previously discussed anthroengineering examples would cease to exist should the term ‘anthroengineering’ not be coined. More importantly, why is it necessary to recognize anthroengineering as its own field?

First, as previously mentioned, names provide identity and symbolic meaning. Should it not be given a unifying name, anthroengineering will remain elusive and ill-defined. In a well-known paper on evolutionary theory, Gould & Vrba [129] present a new word—exaptation—to describe an evolutionary phenomenon. They argue that the existing word ‘adaptation’ is defined and recognized by two criteria and biologists fail to recognize potential confusion between these criteria. Part of the reason for this confusion, they go on to say, is that one of these criteria does not have a distinctive word to describe it. They then propose that the word ‘exaptation’, which had not previously existed, be used for this criterion [129]. By providing a phenomenon with a name, Gould and Vrba took a previously undefined concept and centred it, making it tangible and real. Similarly, while anthroengineering has existed for decades, it has remained abstract and ill-defined. By providing a word to describe this line of work, anthroengineering becomes tangible and real.

Second, providing the name anthroengineering allows for the field to be recognized. This provides a thread to unite researchers working at the intersection of anthropology and engineering, much as the word ‘anthropology’ ties together cultural, linguistic and biological anthropologists, or ‘engineering’ ties together chemical, mechanical and computer engineers. Anthropology and engineering intersect across so many areas of interest that researchers in one area are often ignorant of people working in another (e.g. design anthropologists versus palaeo-biomechanists). The word anthroengineering creates a unifying concept for these researchers and an umbrella under which those anthroengineers can meet with, learn from and work with each other.

Third, the creation of a word and field to describe this line of work creates with it a new way of thinking and new framework, but, unlike interdisciplinary projects, it also creates a permanency. This allows researchers to be trained in this novel way of thinking and apply it with a deeper understanding to new problems in the future. This will then open a new world of potential applications for anthroengineering and enable researchers to ask questions they previously would not have considered.

Once anthroengineering is established and researchers have become fully trained in the field, the questions researchers ask will change. Instead of asking how anthropology or engineering, individually, could address a problem, researchers will ask how anthroengineering can address the problem and—as such—be able to answer it in a more fully informed, comprehensive manner. New questions can be asked, such as:

— How can we leverage anthroengineering to address large problems in the world, such as the UN's SDGs?
— How can we use anthroengineering to better understand how humans have evolved and why modern human biological variation exists in the manner it does?
— How can we leverage that information to better understand how humans are currently evolving in light of technological and societal changes and to address problems associated with racism and other identity-based biases in our technology and societies [130]?
— How can we use advanced modelling techniques to address global problems associated with healthy human ageing?

### 4.1. Creation of a new field

Today, many of the problems facing anthroengineering are the same as those facing interdisciplinary research in general. We recognize the issues facing research and research projects can often be distinct from those facing fields, but, at the time of writing, anthroengineering has almost solely existed at the research level, so it has not yet developed (m)any unique ‘field-level’ problems. As the plights of interdisciplinary research are much discussed, we will provide an overview of some of the main problems facing interdisciplinary research that we have witnessed within anthroengineering. We will further discuss some issues specific to anthroengineering today.

#### 4.1.1. Publishing

Publications are currency in academia. When academics try to demonstrate their impact as researchers, they often total their number of publications, h-index, i10 index and the like for good reason. Publications foster recognition and the institutionalization of research, which in turn feeds back on the infrastructure and capacity of centres and departments, resulting in increased support [1].

Anthroengineers are faced with several difficulties when it comes to publication that plague interdisciplinary research. When making the decision on where to publish, anthroengineers must choose between specialist and generalist journals [131]. Often, their manuscripts do not fit within the narrow remits of specialist journals and would have to change position from a truly transdisciplinary approach to one where the methods/theories from one field are being used to advance the other [132]. Until specialist anthroengineering journals are established, therefore, manuscripts must be published in generalist journals. The risk when publishing in generalist journals is that the paper will not have its desired impact, as the generalist journal may not be regularly read by anthropologists, engineers or fellow anthroengineers. The paper would then miss its target audience.

The most effective way of circumventing this issue is through publication in high-impact generalist journals with large readership bases. But herein lies two dilemmas: (i) high-impact generalist journals tend to have word/page limits, and there is often not enough space to fully explain or discuss the anthropological *and* engineering theories and methods, and (ii) these journals have many submissions and limited publication space. They are, therefore, likely only to publish material they believe will be of interest to a high percentage of their audience, meaning that they can be hesitant to accept and publish papers in untested areas that do not already have a demonstrated readership base.

Further, the editors handling the manuscripts are unlikely to be anthroengineers and are more likely to be either anthropologists or engineers, making it less likely they will be able to grasp fully the impact of the research as part of the work is outside their area of expertise. The same issue occurs when recruiting reviewers for the manuscript [133]. Often, few researchers exist with the expertise to comprehensively review the manuscript. Consequently, more reviewers must be recruited, and it is not uncommon for reviewers to provide conflicting reviews. When conflicting reviews are received by a high-impact journal, the manuscript is often rejected, as the lack of consistency among reviewers is believed to be indicative of an inferior manuscript.

As a result, researchers are required to spend years publishing high-impact research in lower impact generalist journals that may not reach their target audience, and/or moulding their research to reach the narrow remit of the specialist journals. As institutional and funding support are often hinged on the ability to publish in high-impact journals (as this is often used as a metric for the ‘quality’ of research), researchers in interdisciplinary fields must often work much harder to be recognized. Fortunately for anthroengineering, several well-respected journals have been receptive to the publication of anthroengineering manuscripts (e.g. those published by the Royal Society [106,107,134], *Proceedings of the National Academy of Sciences of the United States of America* [12] and *Nature* [58]), but more explicit definition of the field will extend this acceptance.

#### 4.1.2. Funding bodies

Funding is almost as important as publishing in academia, but securing funding for interdisciplinary projects comes with many of the same problems [132,135]. Instead of choosing between specialist journals, researchers are forced to choose between specialist councils (e.g. the Engineering and Physical Sciences Research Council (EPSRC), Natural Environment Research Council (NERC) and Biotechnology and Biological Sciences Research Council (BBSRC) in UK Research and Innovation (UKRI)) or specialist research areas (Biological Sciences, Engineering, International Science and Engineering, and Social, Behavioral, and Economic Sciences in the National Science Foundation (NSF)).

At a time when inter-/multidisciplinary research is heralded as the future of academia [136–138], the narrow focus of councils/research areas makes it complicated to submit interdisciplinary proposals and receive funding. When proposals are submitted to a specific research council/area, the proposal's merit is judged within the expertise of that council/area. While submission of truly interdisciplinary proposals that transcends the boundaries of the research councils/areas can occur through cross-council submissions, councils need to be contacted prior to submission to determine if the proposal is of interest. It often takes months to answer interdisciplinary enquiries, as it requires cross-council conversations, which delay proposal submission.

Once submitted, it is consistently more difficult to be awarded funding for interdisciplinary projects [139], and it is easier to secure funding for projects that combine closely related disciplines than for disparate ones [132]. This, unfortunately, leads to a situation where the more ground-breaking the collaboration is, the harder it is to fund. Lower funding success rates are believed to originate from a bias against interdisciplinary projects. Firstly, interdisciplinary proposals are viewed as higher risk because they do not follow an established path [139]. Secondly, as with journal articles, proposals are often reviewed by reviewers and panels who are ill-equipped to evaluate all parts of the project, making it difficult for them to appreciate the scope and impact of the proposal. They instead only review the portion of the proposal for which they are an expert and are more likely to assign a mediocre or poor score to an interdisciplinary proposal than an intradisciplinary one owing to a poor understanding of the project or the foundational concepts. Having a mix of reviewers who do and do not fully appreciate or understand the project will lead to proposals being rejected, as a lack of consistency between the reviewers is viewed as a problem with the application and not the review process. Additionally, interdisciplinary proposals compete with intradisciplinary ones, which are easier to justify for the funding agent [139].

#### 4.1.3. Institutional support

In the longer term, for anthroengineering—or any other interdisciplinary line of research—to succeed, it must have career-level institutional support. Once interdisciplinary grants are awarded, the resulting projects often include graduate students and/or postdoctoral research associates. While this training expands their knowledge in ways that we recommend, it also leads to the training of a cohort of interdisciplinary researchers who, in the case of anthroengineering, do not fit the classic definitions of anthropology or engineering. They are often not considered ‘real’ anthropologists or ‘real’ engineers. As a result, when it comes time for these individuals to obtain permanent posts, the more interdisciplinary they are, the more difficult it is to obtain a permanent position.

During faculty searches, departments/divisions look for individuals to fill gaps in programme teaching and/or research foci, often hiring candidates who best fit the discipline(s) in which the programme awards degrees. This makes it difficult for truly interdisciplinary researchers to obtain permanent posts: an anthropologist or engineer who has spent their entire career working within the boundaries of their traditional discipline is a much stronger candidate than an anthroengineer. For the long-term success of anthroengineering, high-level institutional support is needed.

#### 4.1.4. Anthroengineering education

In terms of education, institutions need to go a step further than the current practice. To date, all anthroengineering training has been done on an individual level in the laboratory, which requires an inordinate amount of time and effort from the laboratory's principal investigator, and from the individuals independently seeking out formal educations in both anthropology and engineering. Given how different the two disciplines are, this often requires twice the time and money to be educated in anthroengineering, limiting the ability to study anthroengineering to the privileged. Owing to the clear benefits of interdisciplinary research, and the scientific leaps that have been made by anthroengineering research already, we believe that universities should support formally training students as anthroengineers.

The majority of these students will leave academia and enter the private sector. The students trained as anthroengineers will have immediately transferable skills that make them superior on the job market to other anthropologists/engineers seeking employment. For example, a major concern among engineering companies is how to be more socially responsible, while social responsibility is a central theme in anthropology. The anthroengineers entering the job market will have the skills not only to be practising engineers, but also to be more socially responsible than engineers who have not received this training—something that is direly needed [140]. The anthroengineering cohorts will be trained in both anthropology and engineering from the start of their higher education, and, thus, taught to think using interdisciplinary approaches from the start. These anthroengineers will have the ability to see new questions and novel, innovative answers that cannot be imagined by the current generation of anthroengineering.

## 5. Disciplinary culture

The last issue we would like to touch upon with anthroengineering is that of disciplinary culture. In the creation of a new field, we are in the unique position to create the academic culture for the field. A focus of many disciplines, today, is to address the realities of sexism, racism, homophobia, etc., that have become engrained within these disciplines and academia in general and to take the necessary steps to solve these problems [141]. In the establishment of a new field, we can attempt to create a more inclusive academic environment from its inception [142].

When applying to hold the first symposium on anthroengineering at the American Association of Physical Anthropology (AAPA) conference in Cleveland, Ohio, USA, 2019 (Symposium 13—Anthroengineering: a Biological Perspective), we were required to write a 300-word diversity statement. In it, we described our methods for recruiting symposium participants which reflect our vision of anthroengineering:

In recruiting participants for this symposium, we focused on early career researchers and on members of groups frequently underrepresented in research. Consequently, about half of our participants are women, and others are ethnic minorities and members of the LGBTQA[+] community. By recruiting a diverse group of people at an early stage in their careers, we hope to foster an environment of inclusion that connects to and bolsters other such efforts at the AAPAs and in the discipline of biological anthropology generally… [Anthroengineering should value] the contributions of all people, regardless of sex, gender, ethnicity, or sexual orientation, and supports all types of research that combine anthropology and engineering.

In short, our vision for this new field is one of fairness and inclusivity, but anthroengineering will be housed in academic institutions and is born out of two fields which have their own problems. Fortunately, we are in a position where we can observe the issues present in other fields and strive to avoid those issues in this one.

## 6. Conclusion

In this paper, we have presented the concept of anthroengineering, provided examples of how anthroengineering has been used in the past and outline a plan for the future. Importantly, we have argued that anthroengineering should be recognized as its own, independent field: if you did not already believe this, we hope we have made converts out of you.

We cannot wait to see what the future has in store.

## Data accessibility

This article has no additional data.
